# Isolated transient vertigo: posterior circulation ischemia or benign origin?

**DOI:** 10.1186/s12883-017-0894-2

**Published:** 2017-06-14

**Authors:** Tobias F. Blasberg, Lea Wolf, Christian Henke, Matthias W. Lorenz

**Affiliations:** 1 Department of Neurology, Frankfurt University Hospital, Schleusenweg 2-16, 60528 Frankfurt am Main, Germany; 2 Department of Neurology, Helios HSK Wiesbaden, Ludwig-Erhard-Straße 100, 65199 Wiesbaden, Germany; 3 Department of Neurology, Krankenhaus Nordwest, Steinbacher Hohl 2-16, 60488 Frankfurt/Main, Germany

**Keywords:** Cerebrovascular, Stroke, Transient, Transient ischemic attack, Vertigo

## Abstract

**Background:**

Isolated transient vertigo can be the only symptom of posterior circulation ischemia. Thus, it is important to differentiate isolated vertigo of a cerebrovascular origin from that of more benign origins, as patients with cerebral ischemia have a much higher risk for future stroke than do those with ‘peripheral’ vertigo. The current study aims to identify risk factors for cerebrovascular origin of isolated transient vertigo, and for future cerebrovascular events.

**Methods:**

From the files of 339 outpatients with isolated transient vertigo we extracted history, clinical and technical findings, diagnosis, and follow-up information on subsequent stroke or transient ischemic attack (TIA). Risk factors were analyzed using multivariate regression models (logistic or Cox) and reconfirmed in univariate analyses.

**Results:**

On first presentation, 48 (14.2%) patients received the diagnosis ‘probable or definite cerebrovascular vertigo’. During follow-up, 41 patients suffered stroke or TIA (event rate 7.9 per 100 person years, 95% confidence interval (CI) 5.5–10.4), 26 in the posterior circulation (event rate 4.8 per 100 person years, 95% CI 3.0–6.7). The diagnosis was not associated with follow-up cerebrovascular events. In multivariate models testing multiple potential determinants, only the presentation mode was consistently associated with the diagnosis and stroke risk: patients who presented because of vertigo (rather than reporting vertigo when they presented for other reasons) had a significantly higher risk for future stroke or TIA (*p* = 0.028, event rate 13.4 vs. 5.4 per 100 person years) and for future posterior circulation stroke or TIA (*p* = 0.044, event rate 7.8 vs. 3.5 per 100 person years).

**Conclusions:**

We here report for the first time follow-up stroke rates in patients with transient isolated vertigo. In such patients, the identification of those with cerebrovascular origin remains difficult, and presentation mode was found to be the only consistent risk factor. Confirmation in an independent prospective sample is needed.

## Background

Vertigo is a frequent reason for emergency presentation [1–5] with manifold causes [3, 4, 6–8]. If focal neurological signs or symptoms occur, transient ischemic attack (TIA) may be diagnosed; otherwise cerebral ischemia seems unlikely [6–8]. In unselected samples, (proven) cerebrovascular cause in isolated vertigo is rare [9] and risk for future stroke is low [10], however higher than in other emergency patients [11], in particular when vascular risk factors (VRFs) are present [11]. In the last decade, increasing evidence has been put forward to show that posterior circulation ischemia can present with isolated vertigo without focal signs [9, 12]. Even more disconcerting are findings from the OxVasc study: 22% of posterior circulation stroke patients reported subtle transient neurological symptoms in the 90 days preceding their stroke, most frequently vertigo [13]. To preclude future strokes it is crucial to identify those patients whose vertigo episode was a subtle TIA.

For the clinician, there are two typical situations: the patient with acute onset, ongoing vertigo, and the patient free of symptoms on presentation, who reports (often multiple) transient episodes of vertigo. On a population basis, the lifetime prevalence of the latter is at least as high as that of the former [5]. The former patient is usually seen in a hospital emergency department, whereas the latter may consult a practitioner or an outpatient clinic. In symptomatic emergency patients we have the opportunity to notice subtle clinical (mainly neuro-ophthalmological) findings that may give important clues, as reflected by the HINTS algorithm [14], and – if stroke is likely – acute magnetic resonance imaging (MRI) including diffusion-weighted imaging (DWI) can often prove, but rarely exclude, this diagnosis [15–21]. In outpatients with recurrent transient vertigo, neuro-ophthalmological findings during the attack usually resolve unseen and other information may be lost or biased by recall. DWI may help only when residual microinfarctions persist; mostly MRI is negative in this situation [17].

The identification of patients with cerebrovascular cause of recurrent transient isolated vertigo remains an unsolved clinical problem. The purpose of the current study was to find determinants of cerebrovascular origin, find risk factors for future stroke, and – if possible - construct a predictive model to help guide the diagnosis in patients who presented with isolated transient vertigo in a tertiary cerebrovascular outpatient unit.

## Methods

We screened the patient files of our department’s neurovascular clinic (1 July 2007 to 30 June 2014) for adults who reported isolated vertigo without focal neurological symptoms (dysarthria, focal weakness, sensory symptoms, limb ataxia, diplopia and hemianopia; unsteady gait was allowed). We extracted history, clinical findings, and technical results. The diagnosis (made by a neurologist in training (third year or later) and a consultant with neurovascular specialization) in the initial report was categorized as ‘definite’, ‘probable’ or ‘improbable cerebrovascular vertigo’.

Follow-up information on subsequent stroke or TIA was obtained from the files and a telephone interview. For each endpoint, the observation time was censored at the time of an endpoint event (whereas follow-up was continued for other endpoints), or at the end of a patient’s follow-up.

Associations between clinical and technical variables, diagnosis and follow-up events were addressed with multivariate logistic and Cox regression models, and confirmed with the Kaplan Meier Log rank test and univariate logistic and Cox regression. To limit the number of statistical tests, a stepwise analysis strategy was defined a priori. Although originally planned, we did not attempt to construct a predictive model, as we found no consistent risk factors. All statistical calculations were made with SPSS® (IBM Inc., Armonk, NY, USA).

## Results

From the electronic files of 4714 outpatient contacts, we identified 1355 patient contacts with the relevant keywords and 339 eligible patients (Table 1); 183 (54%) patients had two or more modifiable risk factors and 215 (63%) had coronary, cerebral or peripheral atherosclerosis.

**Table 1:** Baseline characteristics of patients

	Total population (*n* = 339)
Age, years (mean ± SD)	63.4 ± 12.7
Female gender, n (%)	143 (42.2)
Male gender, n (%)	196 (57.8)
Hypertension, n (%)	247 (72.9)
Diabetes, n (%)	74 (21.8)
Smoking, n (%)	77 (22.7)
Hypercholesterolemia, n (%)	125 (36.9)
Hypertriglyceridemia, n (%)	35 (10.3)
Number of modifiable VRFs, #: n (%)	
(Including hypertension, diabetes, smoking, hypercholesterolemia, and hypertriglyceridemia)	0: 49 (14.5)
	1: 107 (31.6)
	2: 123 (36.3)
	3: 37 (10.9)
	4: 21 (6.2)
	5: 2 (0.6)
Coronary heart disease (CHD), n (%)	65 (19.2)
Peripheral arterial occlusive disease (AOD), n (%)	36 (10.6)
Prevalent Stroke or TIA (CVD), n (%)	177 (52.2)
Stroke or TIA in the last 6 months, n (%)	38 (11.2)
At least one vessel disease (CHD, AOD or CVD), n (%)	215 (63.4)

On initial contact 187 (55.2%) patients came to see a neurologist because of vertigo, 152 for other reasons (mainly routine follow-up of known cerebrovascular disease), but reported transient isolated vertigo when asked about new symptoms. Most vertigo episodes lasted less than a minute, details are summarized in Table 2. Neurological examination was unremarkable in 193 (56.9%) patients. Gait ataxia was present in 88 patients (26.0%, prevalent conditions excluded). In 112 patients, the Epley manoeuvre was performed on presentation and was suggestive of benign paroxysmal positional vertigo (BPPV) in 20 (17.9%) patients. Head impulse test was never documented, presumably because the vertigo had subsided by the time of presentation in all cases. Caloric vestibular testing was done in only 15 cases and showed under-excitability in three cases (all diagnosed as vestibular neuritis), and over-excitability in one case (diagnosed as benign positional vertigo). Due to the high proportion of missing information, we did not attempt statistical calculations on caloric testing. Menière’s disease and labyrinthitis were not diagnosed in this cohort.

**Table 2:** Properties of vertigo

	Total population (*n* = 339)
Type of vertigo^a^	
Illusion of rotational movement, n (%)	120 (35.4)
Illusion of swaying movement, n (%)	132 (38.9)
Unclassifiable, n (%)	101 (29.8)
Vertigo frequency	
Median, range (n/week)	5/week, 1/week – 35/week
Below median, n (%)	40 (35.6 of noted)
Median or higher, n (%)	73 (64.4 of noted)
Not noted, n (%)	226 (66.7)
Vertigo duration	
Median, range (seconds)	< 60s, 1 s – 10.800 s
Below median, n (%)	84 (56.4 of noted)
Median or higher, n (%)	65 (43.6 of noted)
Not noted, n (%)	190 (56.0)
Vertigo trigger^a^	
Spontaneous, n (%)	200 (59.0)
Turning the head, n (%)	61 (18.0)
Other change in body position, n (%)	123 (36.3)
Orthostatic stress, n (%)	37 (10.9)
Blood pressure-lowering situation, n (%)	40 (11.8)

^a^Classification not exclusive as some patients reported multiple types

Brain imaging included cranial computed tomography (CT) in 66 (19.5%) and in 150 (44.2%) patients; CT or MRI showed definite new cerebral infarction in eight (2.4%) patients. Vessel imaging included duplex sonography in 327 (96.4%), CT angiography in ten (2.9%), MR angiography in 57 (16.8%) and digital subtraction angiography (DSA) in 14 (4.1%) patients. Vessel findings (Table 3) were assembled from all available information.

**Table 3:** Findings in cervical and cerebral arteries with focus on posterior circulation (*n* = 339)

	Right	Left
Subclavian artery		
Stenosis, n (%)	23 (6.8)	37 (10.9)
No stenosis, n (%)	198 (58.4)	184 (54.3)
Not examined, n (%)	118 (34.8)	118 (34.8)
Vertebral artery V0-V4		
Stenosis ≥50%, n (%)	76 (22.4)	71 (20.9)
Stenosis <50%, n (%)	26 (7.7)	15 (4.4)
No stenosis, n (%)	225 (66.4)	241 (71.1)
Not examined, n (%)	12 (3.5)	12 (3.5)
Vertebral artery V0-V1		
Stenosis ≥50%, n (%)	41 (12.1)	39 (11.5)
Stenosis <50%, n (%)	18 (5.3)	7 (2.1)
No stenosis, n (%)	265 (78.2)	281 (82.9)
Not examined, n (%)	15 (4.4)	12 (3.5)
Vertebral artery V2		
Stenosis ≥50%, n (%)	22 (6.5)	24 (7.1)
Stenosis <50%, n (%)	3 (0.9)	4 (1.2)
No stenosis, n (%)	302 (89.1)	299 (88.2)
Not examined, n (%)	12 (3.5)	12 (3.5)
Vertebral artery V3-V4		
Stenosis ≥50%, n (%)	42 (12.4)	37 (10.9)
Stenosis <50%, n (%)	9 (2.7)	7 (2.1)
No stenosis, n (%)	274 (80.8)	282 (83.2)
Not examined, n (%)	14 (4.1)	13 (3.8)
Basilar artery		
Stenosis ≥50%, n (%)	27 (8.0)	
Stenosis <50%, n (%)	16 (4.7)	
No stenosis, n (%)	283 (83.5)	
Other findings		
Basilar head aneurysm, n (%)	2 (0.6)	
Megadolichobasilaris, n (%)	1 (0.3)	
Not examined, n (%)	13 (3.8)	
Internal carotid		
Stenosis ≥70% (NASCET), n (%)	32 (9.4)	28 (8.3)
Stenosis <70%, n (%)	30 (8.8)	50 (14.7)
No stenosis, n (%)	265 (78.2)	248 (73.2)
Other finding		
Pseudoaneurysm, n (%)	0 (0.0)	1 (0.3)
Not examined, n (%)	12 (3.5)	13 (3.8)
Intracranial artery^a^		
Stenosis ≥50%, n (%)	4 (1.2)	
No stenosis or <50%, n (%)	324 (95.6)	
Other findings		
Right PICA occlusion, n (%)	1 (0.3)	
Untreated right MCA aneurysm, n (%)	1 (0.3)	
Coiled AcomA aneurysm, n (%)	1 (0.3)	
Not examined, n (%)	11 (3.2)	
Any vertebral or basilar artery		
Stenosis ≥50%, n (%)	119 (35.1)	
No stenosis or <50%, n (%)	209 (61.7)	
Not examined, n (%)	11 (3.2)	
Bilateral vertebral or basilar artery		
Stenosis ≥50%, n (%)	57 (16.8)	
No stenosis or <50%, n (%)	271 (79.9)	
Not examined, n (%)	11 (3.2)	
Any pathologic finding (any degree)^b^		
Pathologic finding, n (%)	205 (60.5)	
Normal, n (%)	123 (36.3)	
Not examined, n (%)	11 (3.2)	
Any stenosis ≥ 50%^b^		
Stenosis ≥50%, n (%)	191 (56.3)	
No stenosis or <50%, n (%)	137 (40.4)	
Not examined, n (%)	11 (3.2)	

^a^Other than basilar artery

^b^Extra- or intracranial

### Diagnosis and follow-up

In the medical report from the initial contact, vertigo was considered as ‘definitely cerebrovascular’ in 19 (5.6%) patients and ‘definitely or probably cerebrovascular’ in 48 (14.2%) patients.

In 214 (63.1%) patients our files included at least one further presentation after the initial contact. The remaining 125 patients were contacted and 26 did not respond or refused to participate in the survey. Ninety-nine patients gave their consent and provided follow-up information (response rate 79.2%). Overall, we obtained follow-up information from 289 of 339 patients (85.3%, follow-up period 3 days to 7.7 years, 563 person years). The number of endpoint events and the resulting event rates are shown in Table 4.

**Table 4:** Stroke endpoints during follow-up

	Number of events	Event rate per 100 person years (95% CI)
Any Stroke	22	4.0 (2.3–5.6)
Posterior circulation stroke	12	2.1 (0.9–3.3)
Any stroke or TIA	41	7.9 (5.5–10.4)
Posterior circulation stroke or TIA	26	4.8 (3.0–6.7)

### Determinants of the clinical diagnosis

Age was positively associated with a higher risk for the diagnosis ‘definite or probable cerebrovascular vertigo’ (multivariate and univariate *p* = 0.024). Presentation because of vertigo was positively related with the diagnosis ‘definite cerebrovascular vertigo’ (multivariate *p* = 0.013; univariate *p* = 0.014). Patients with fewer than five vertigo attacks per week were more likely to be diagnosed with ‘definite or probable cerebrovascular vertigo’ (multivariate and univariate *p* < 0.001). Patients with bilateral vertebral stenosis or basilar stenosis and patients with any stenosis were more likely to be diagnosed with ‘definite or probable cerebrovascular vertigo’ (multivariate *p* = 0.047 and *p* = 0.048; univariate *p* < 0.001 each).

### Determinants of future cerebrovascular events

**Fig. 1:**
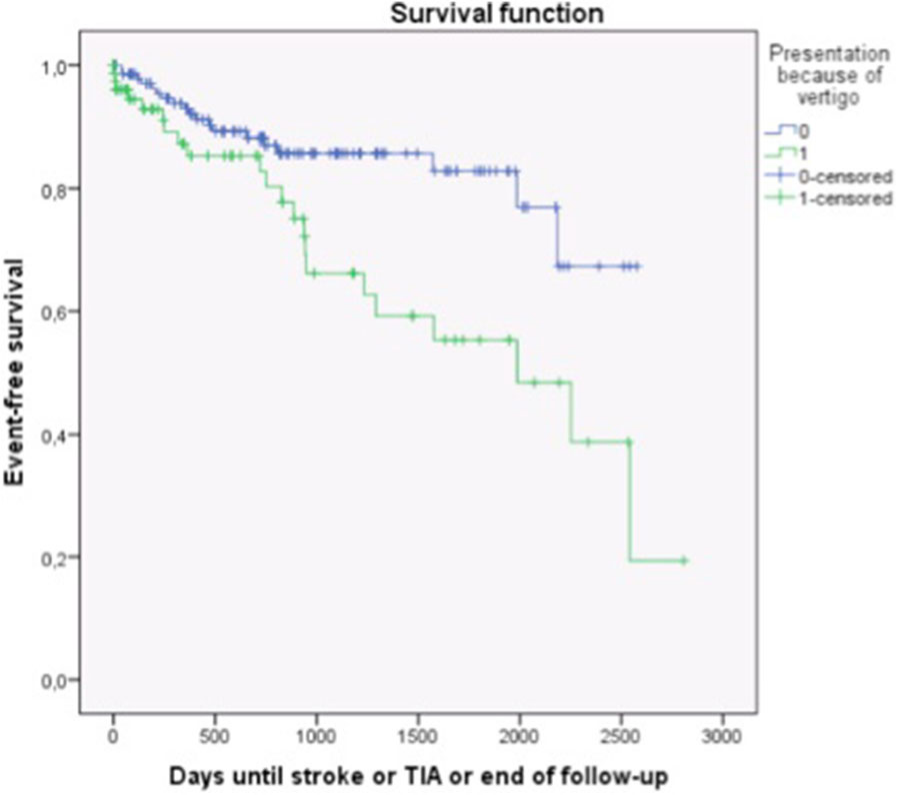
Kaplan Meier plot of the endpoint ‘any stroke or TIA’, stratified by presentation mode. Event rates (presentation because of vertigo (1) vs. other reasons (0)): 13.4 (95% CI 7.8-19.0) vs. 5.4 per 100 person years (95% CI 3.0-7.8)

In patients who presented because of vertigo, the future risk for stroke or TIA was significantly higher than in patients who presented for other reasons (multivariate *p* = 0.028, univariate *p* = 0.005, adjusted HR 2.07 (1.11–3.84); Fig. 1). When tested for the other endpoints (univariate), the reason for presentation was seen to be a significant determinator of stroke or TIA in the posterior circulation (*p* = 0.044, event rate 7.8 vs. 3.5 per 100 person years), but not of ‘any stroke’ or ‘posterior circulation stroke’.

Patients whose vertigo was provoked by a change in body position had a smaller risk for posterior circulation stroke or TIA (multivariate *p* = 0.037; univariate *p* = 0.017; event rates 1.9 (95% CI 0.0–3.8) vs. 5.4 per 100 person years (3.9–9.4)). Patients with any stenosis were less likely to suffer stroke (multivariate *p* = 0.032; univariate *p* = 0.013, event rate 2.6 vs. 7.5 per 100 person years). The diagnosis was not associated with the risk for future cerebrovascular events (tested in eight univariate Cox regression models, all *p* > 0.3).

## Discussion

Aim of the present study was to find determinants to identify those with subtle TIAs among patients with isolated transient vertigo, and to identify risk factors for future stroke or TIA during follow-up. Our sample of 339 persons was a high-risk population with multiple VRFs and a high proportion of vascular organ damage. Usually, patients are referred to this unit because the referring physician believes there is a problem with the cervical or cerebral arteries.

A somewhat comparable population may be patients presenting with acute onset vertigo, although there are important differences: their vertigo episode may be singular (all recurrent in our cohort), possibly more severe (as acute patients called an ambulance immediately, whereas our patients consulted their physician days or weeks later), and presumably longer (in acute patients, the symptoms are mostly still present on arrival at the hospital emergency department).

In comparison with the literature data on acute vertigo, absolute follow-up stroke rates were higher in our cohort (any stroke in 4% of the cohort per year) as compared to 1.4% per year in patients who presented with ‘non-stroke dizziness’ to an emergency department in Texas, USA [10], and 1.7% per year in patients hospitalized for vertigo in Taiwan [11]. This discrepancy may be largely explained by the high risk factor load in our cohort: a subgroup of Taiwanese patients hospitalized for vertigo with the most VRFs had a 3.5% annual stroke rate [11], which is very similar to our results. The proportion of patients with stroke as the cause of the initial episode was 0.7% in the Texas cohort [9], whereas in our cohort stroke or TIA was the definite cause in 5.6% and the probable or definite cause of vertigo in 14.2% of our cohort. Again, the main reason for this discrepancy is most likely the large number of VRFs among our patients.

In the search for determinants of the diagnosis *cerebrovascular vertigo*, we identified age and precerebral artery stenosis, which were both expected. Unexpectedly, both these determinants were inconsistent for the stroke endpoints: here age was not identified at all as a predictor, and stenosis of any cervical or cerebral vessel was even negatively associated with future stroke. For the diagnosis on initial presentation, these two ‘obvious’ risk factors may have influenced the neurologist’s judgment. The frequency of vertigo attacks was predictive for the diagnosis ‘definite or probable cerebrovascular vertigo’, where lower frequency was associated with cerebrovascular origin. As many uniform episodes are difficult to explain as TIAs, this factor may also have influenced the judgment of the diagnosing neurologist.

‘Reason for presentation’ was the only determinant that was consistently and significantly associated with the diagnosis ‘cerebrovascular vertigo’ and with future posterior circulation stroke and TIA. It is possible that vertigo, which is due to a subtle brainstem or cerebellar TIA, may be more intense or impressive than vertigo of other causes, thus persuading the patient or his primary care physician to make an urgent clinic appointment.

A very interesting influential factor was identified only in the endpoint analysis: the provocation factor ‘changes in body position’, which was associated with lower stroke risk. This association may tell us that in our cohort peripheral positional vertigo (for example BPPV or benign disabling vertigo [22]) may be more frequent than (ischemic) central positional vertigo (e.g. pseudo-BPPV in vermis stroke/TIA [23]). The lack of an association between ‘head rotation’ as a trigger of vertigo and future stroke can mean either that vertigo caused by functional vertebral artery compression [22] is rare in our patients or – if it occurs – that it rarely causes stroke.

The most surprising result of our work is that the judgment of the vascular neurologist was not correlated with future stroke risk. At first glance, this challenges our view of the world: are our sophisticated pathophysiological considerations out of sync with reality? A more comfortable explanation may be that the originally elevated stroke risk for patients correctly classified as ‘subtle vertigo TIA’ was counteracted by the risk factor management we subsequently recommended. Such a hypothetical treatment bias might even explain the inverse association between ‘any stenosis’ and future stroke, as escalations of risk factor management (e.g. tightening low-density lipoprotein cholesterol (LDLc) goals) may be triggered by finding stenoses.

What can we learn from these data for the management of our patients? First, we must be careful not to over-interpret these results, as they are explorative and require external validation. Given our sparse and somewhat inconsistent findings, we refrained from constructing an originally planned predictive model. On the basis of the risk factor ‘reason for presentation’ we may consider doing an MRI in patients with isolated transient vertigo, who are worried enough to see the doctor because of this symptom. A clinical benefit of this measure has yet to be proven. Replication of this study in a prospective design may yield the necessary information for constructing a predictive model and developing a refined clinical algorithm.

### Limitations

Some limitations of this study arise from the retrospective design. For example, some important variables characterizing the vertigo (frequency and duration) were incompletely documented (33% and 44%, respectively). A complete documentation of these variables in a prospective setting may yield interesting results. Furthermore, in only 60% of the patients was a pathologic finding detected with vessel imaging, which is unexpectedly low in such a high-risk cohort. In particular, low-grade stenoses (<50% or <70%) were relatively rare. Possibly minor vessel changes were under-reported, as the ultrasound examiner may have focused on ‘relevant’ high-grade findings. However, as our analysis focused on high-grade stenoses (≥50% or ≥70%), it is unlikely that this caused relevant bias.

Importantly, the nature of our analyses was explorative, as many determinants were tested for multiple dependent variables. Despite provisions to reduce the number of statistical tests (see Methods section), 15 determinants were tested for six dependent variables, resulting in 90 primary tests. A Bonferroni correction would have reduced the *p* value to 0.0005, which is an unlikely *p* value in multivariate models in a cohort of this size. Therefore, all results have to be interpreted with caution.

## Conclusions

We here reported for the first time follow-up stroke rates in patients with isolated transient vertigo. Identifying patients with cerebrovascular vertigo remains difficult. Presentation mode (patients who presented because of vertigo) was found to be the only consistent risk factor for cerebrovascular vertigo and future risk for stroke or TIA. However, the clinical benefit of this finding may be limited. Confirmation in an independent prospective sample is needed.

## Abbreviations

BPPV: Benign paroxysmal positional vertigo
CI: Confidence interval
CT: Computed tomography
DSA: Digital subtraction angiography
DWI: Diffusion-weighted imaging
LDLc: Low-density lipoprotein cholesterol
MRI: Magnetic resonance imaging
TIA: Transient ischemic attack
VRFs: Vascular risk factors
